# Student engagement and learning outcomes: an empirical study applying a four-dimensional framework

**DOI:** 10.1080/10872981.2023.2268347

**Published:** 2023-10-08

**Authors:** Xiaoming Xu, Zehua Shi, Nicolaas A. Bos, Hongbin Wu

**Affiliations:** a International Institute of Medicine, Zhejiang University, Yiwu, Zhejiang, China; b The Fourth Affiliated Hospital, Zhejiang University School of Medicine, Yiwu, Zhejiang, China; c Center for Educational Development and Research in health sciences (CEDAR), LEARN, University Medical Center Groningen, University of Groningen, Groningen, the Netherlands; d National Centre for Health Professions Education Development/Institute of Medical Education, Peking University, Beijing, China

**Keywords:** Student engagement, learning outcomes, medical education, higher education, national survey

## Abstract

**Introduction:**

This study applies Reeve’s four-dimensional student engagement framework to a medical education context to elucidate the relationship between behavioral, emotional, cognitive, and agentic engagement and learning outcomes. Meanwhile, we categorize learning outcomes in knowledge and skills, and added taxonomies to the cognitive education objectives for the knowledge part, including memorization, comprehension, and application.

**Methods:**

We used the China Medical Student Survey to investigate student engagement, and combined it with the Clinical Medicine Proficiency Test for Medical Schools results as a standardized measurement of learning outcomes. We performed multivariate regression analyses to delve into the effectiveness of different types of student engagement. Moreover, we evaluated the moderating roles of gender and the National College Entrance Examination (NCEE) within the relationships between student engagement and learning outcomes.

**Results:**

We observed that emotional engagement is most effective in promoting learning outcomes in basic medical knowledge and basic clinical skills. Emotional engagement and cognitive engagement could effectively contribute to learning outcomes in all three aspects of basic medical knowledge. In contrast, behavioral and agentic engagement showed negative effects on learning outcomes. Besides, we found that the results of the NCEE played a positive moderating role.

**Conclusion:**

This study provides robust evidence for the effectiveness of emotional engagement and cognitive engagement in promoting learning outcomes. Whereas behavioral and agentic engagement may not be good predictors of learning outcomes in macro-level general competence tests. We suggest a combined effort by students and institutions to promote student engagement and bridge the distance between general competency tests and daily learning activities.

## Introduction

Student engagement is a pivotal topic in higher education research, policy, and practice [1,2]. Student engagement has been defined as ‘the interaction between the time, effort and other relevant resources invested by both students and their institutions intended to optimize the student experience and enhance the learning outcomes and development of students and the performance, and reputation of the institution’ [3]p3). The definition of student engagement emphasizes the shared responsibility of both students and institutions for the quality of student learning, which means that student development needs to come from the combined efforts of students themselves and a conducive learning environment of the institutions [3–6]. Moreover, student engagement has been generally considered an essential predictor of students’ learning and personal development [4,7,8]. The effectiveness of student engagement has received extensive attention such as in students’ school satisfaction [6,9], and in learning outcomes and academic achievements [7,10,11]. Nevertheless, we observe that not all of the literature was using a coherent framework for student engagement. To be specific, when trying to look into the multidimensional construct of student engagement, one may get confused by the different categorical information available, making it difficult to find concrete suggestions for guidance in educational practice [12,13].

Among the literature, several prevailing frameworks of student engagement received extensive attention. Finn [14] contributed to one of the first attempts to classify student engagement to explain the dropout issue and proposed two engagement variables: identification and participation. Skinner, Kindermann, & Furrer [15] experimented with a motivational perspective and presented four engagement components: behavioral engagement, behavioral disaffection, emotional engagement, and emotional disaffection. Another seminal work from Fredricks, Blumenfeld, & Paris [16] helped build consensus on the student engagement framework, in which they described student engagement in three forms: behavioral, emotional, and cognitive. Stories have begun to build on Fredricks et al. [16] advanced framework, and one notable work came from Reeve & Tseng [8], which added an agency dimension to the original three-dimensional framework. The newly-add agentic engagement complements students’ proactive attempts and their interactivity with the institution [8,13,17]. To consider student engagement comprehensively, this study adopts the refined four-dimensional framework containing *behavioral*, *emotional*, *cognitive*, and *agentic engagement*, and investigated student engagement in learning activities using this structural framework.

To briefly explain the four distinct and highly inter-correlated engagement dimensions: *behavioral engagement* means students typically adhere to behavioral norms such as attendance and participation, and demonstrate no disruptive or negative behavior; *emotional engagement* means students experience emotional responses such as interest, enjoyment, or a sense of belonging; *cognitive engagement* means students would seek to go beyond the requirements and would use sophisticated learning strategies; and *agentic engagement* means students proactively attempt to enrich their learning experience [3,8,16,18,19].

The effectiveness of each student engagement dimension has been discussed in various contexts. More specifically, behavioral engagement in classroom contributes to students’ school success [20]; emotional engagement may play a positive role in predicting students’ satisfaction [9]; cognitive engagement could directly predict learning outcomes [21]; and agentic engagement could independently predict students’ achievement and motivation [8,17]. Furthermore, relevant research has especially examined the effectiveness of student engagement dimensions on academic achievement. Through doing a meta-analysis of 69 independent studies, Lei, Cui, & Zhou [22] found that behavioral engagement, emotional engagement, and cognitive engagement all positively correlated with academic achievement. Reeve et al. [23] conducted two empirical studies with Korean secondary school students and showed that agentic engagement explained independent variance of course achievement and academic progress after controlling for other dimensions. In contrast, a different study on university students in Dominican Republic [24] showed that it was cognitive engagement and emotional engagement (not agentic engagement) that had a specific positive effect on academic achievement. While looking into the Health Professional Education context, the findings on the relationship between student engagement and learning outcomes have been inconsistent [25,26]. For instance, Rotgans, et al. [27] reported cognitive engagement as a significant predictor of academic achievement when examining cognitive engagement specifically. However, other studies found student engagement may have poor correlations with higher-order skill proficiency [28,29]. The variable research findings and unclear dimensions of student engagement call for more empirical studies in different research contexts to provide evidence for student engagement within a unified research framework [13]. If research investigates only some of these dimensions of student engagement, or treats student engagement as a holistic concept, it is unclear whether all dimensions of engagement play the same role, and how we can apply student engagement in more practical ways [4,30].

Meanwhile, capitalizing on the effectiveness of student engagement requires assessing learning outcomes rigorously and objectively and examining the relationships in-between [5,11]. Considerable social science research often used self-reported data to estimate students’ learning outcomes [5,31,32]. Although the self-reported information could be valid under certain conditions, learning outcomes are measured on an individual basis rather than on a standardized basis [5,31]. The self-reported learning outcomes could be biased due to internal factors (e.g., self-image), interpersonal factors (e.g., relationships with peers), and external and perceived criteria (e.g., culture) [33]. Moreover, the application of a taxonomy that categorizes cognitive education objectives could largely increase the communicability and comprehensiveness of the learning outcomes [34,35]. Research that incorporates cognitive education objective categories into learning outcomes is also scarce in relevant student engagement articles. Consequently, we lack detailed information on how student engagement as a multidimensional structure contributes to different parts of learning outcomes.

To overcome the bias from self-reported learning outcomes and bring categorized cognitive education objectives into the investigation, this study used learning outcome data from a nationally standardized examination, *the Clinical Medicine (BSc) Proficiency Test for Medical Schools* in China (hereafter referred to as *the Proficiency Test*). The Proficiency Test has been used to assess whether medical undergraduates have knowledge of medical humanities, basic medical knowledge, and basic clinical skills before entering the clinical rotation. Similar to the setting in the United States Medical Licensing Examination (USMILE) Step 2, the Proficiency Test includes the Basic Medical Knowledge Examination and Basic Clinical Skills Examination, which made up the two parts of learning outcomes. In addition to the categorizing of knowledge and skills, the Basic Medical Knowledge Examination pertains to three cognitive levels: memorization, comprehension, and application [36,37]. Within the development of assessment questions in the Basic Medical Knowledge Examination, categories of memorization, comprehension, and application have been used [36].

Additionally, noticing that the learning outcomes could be influenced by various elements other than student engagement [6,7,11,38], we took gender, residence, parents’ education duration, parents’ occupation, whether having medical worker(s) in the family, and students’ National College Entrance Examination (NCEE) results into consideration and added those characteristics as controls. Furthermore, the NCEE, representing students’ pre-college experience, has been regarded as an effective predictor of students’ university performance [39]. Besides, gender has been considered an important moderating factor in relevant studies [6,9]. Therefore, we also investigated whether the relationships between student engagement and learning outcomes are conditionally depending on the NCEE results and gender.

To conclude, this study tried to answer the research questions of: 1) *To what extent does student engagement (behavioral, emotional, cognitive, and agentic) relate to the learning outcomes?* 2) *How do the categories of learning outcomes (memorization, comprehension, and application) relate to each dimension of student engagement?* 3) *Whether the relationships between student engagement and learning outcomes vary depending on students’ demographic background (Gender and NCEE result)?* The use of standardized learning outcomes in medical education in this study could also bridge the gap in higher education where standardized learning outcomes are rarely used. Besides, as medicine in China is one of the professions in which Chinese high school students enter higher education after going through the National College Entrance Examination (NCEE), the findings of this study could also provide robust empirical evidence for research, policy, and practice in higher education in general.

## Methods

### Sample and data collection

Data collection for this study came from two datasets. We used China Medical Student Survey (CMSS) 2021 to investigate student engagement, and used the Proficiency Test result as the standardized measurement of learning outcomes for these medical students. The CMSS is a national-wide survey dedicated to Basic Medical Education in China by the National Centre for Health Professions Education Development. It investigates medical undergraduates about a wide range of demographic characteristics, student engagement, learning process, and perceptions towards medical education programs. The CMSS is the largest and most detailed survey for medical education in China. In 2021, 121 medical schools across 30 provinces in China completed the CMSS with a response rate of 64.6%. The Proficiency Test is a unified examination in China jointly organized and administered by the National Medical Examination Center and National Center for Health Professions Education Development. Medical undergraduates take the Proficiency Test at the end of the fourth year of undergraduate medical education before they enter the clinical rotation phase. We extracted students’ proficiency test scores from the database directly and matched them with the student IDs instead of asking students to self-report their learning outcomes. The participants of this study are fourth-year medical undergraduates who have filled in the CMSS and finished taking the Proficiency Test.

### Measures

This study has a sample size of 13,010, with 5771 (44%) males and 7239 (56%) females. Besides, 5848 participants (45%) come from rural area and 7162 (55%) come from urban area. The average NCEE score of all participants is 537.70. Table 1 shows the descriptive statistics we used in this study. We selected the student engagement items by applying the theoretical framework from Reeve [18] to the medical education content and modified the items in current literature intending to evaluate medical students’ student engagement perceptions [8,18,40,41]. Afterward, we examined the validity and reliability by calculating each dimension’s Cronbach’s α and doing factor analysis. More concretely, we selected five items under the behavioral engagement dimension [8,18]; three items under the emotional engagement dimension [18,40]; three items under the cognitive engagement dimension [8,18]; and three items under the agentic engagement dimension [8,18,19,41] (see Table 1). In consideration of medical students’ clinical training, we put two components into behavioral engagement, which include behavioral clinical engagement (B1, B2, B3) and behavioral classroom engagement (B4, B5). Participants were asked to respond to items on a five-point Likert scale (from 1 - strongly disagree to 5 - strongly agree). The original questionnaire was administered in Chinese and has been translated into English when reporting the results in this paper.

**Table 1.** Descriptive statistics.

	Participant characteristics	Mean	SD	Observations
1	Gender (Male)	0.44	0.50	13010
2	Residence (Rural area)	0.45	0.50	13010
3	Father’s education duration	10.73	3.77	13010
4	Mother’s education duration	9.60	4.17	13010
5	Father’s occupation ISEI	33.04	18.89	13010
6	Mother’s occupation ISEI	29.99	17.14	13010
7	Having medical worker(s) in the family	0.27	0.45	13010
8	NCEE result	537.70	52.00	13010
	Independent Variables: Student engagement			
	Behavioral engagement	Cronbach’s α: 0.69		
B1	Participating in teaching rounds	3.87	1.04	13010
B2	Participating in case-reporting	3.39	1.08	13010
B3	Engaging in clinical operations	3.29	1.11	13010
B4	Doing academic-related presentations	2.68	0.98	13010
B5	Participating in cooperative group learning or discussions	3.28	0.86	13010
	Emotional engagement	Cronbach’s α: 0.79		
E1	I am interested in my field of study	3.68	0.79	13010
E2	I want my future career to be closely related to my profession	3.92	0.79	13010
E3	I am passionate about learning	3.55	0.76	13010
	Cognitive engagement	Cronbach’s α: 0.75		
C1	When I encounter difficulties in my studies, I can usually think of some solutions to deal with them	3.68	0.72	13010
C2	I have participated as an active learner with responsibility for my own learning	3.54	0.81	13010
C3	I have assessed my own competence	3.58	0.81	13010
	Agentic engagement	Cronbach’s α: 0.78		
A1	My feedback has been taken into account in curriculum development	3.39	0.91	13010
A2	I have been involved formally and/or informally in peer teaching (explaining the appropriate knowledge to my peers)	3.49	0.83	13010
A3	I have engaged in peer assessment	3.41	0.88	13010
	Dependent Variables: Learning outcomes			
D1	Basic Medical Knowledge Examination	190.64	34.17	13010
D1.1	Memorization	42.39	8.29	13010
D1.2	Comprehension	56.99	9.45	13010
D1.3	Application	91.24	18.34	13010
D2	Basic Clinical Skills Examination	80.32	9.72	13010

As for the learning outcome measures, the Basic Medical Knowledge Examination is conducted in the form of a Computer-aided Test. It consists of 20–25% for memorization, 25–30% for comprehension, and 45–50% for application questions. The Basic Clinical Skills Examination is conducted in the form of an Objective Structured Clinical Examination with evaluating students’ medical history taking, physical examination, and basic operative skills. Both the Basic Medical Knowledge Examination and the Basic Clinical Skills Examination are scored out of 100. We provided sample items of the Proficiency Test in the supplementary material (ST1).

### Data analysis

To facilitate the correlation analysis between student engagement and learning outcomes, we first applied Principal Component Factor analysis to each student engagement dimension for clear interpretability. The factor analysis results provided evidence that supported that there are two components within *behavioral engagement* including *behavioral clinical engagement* and *behavioral classroom engagement* while *emotional engagement*, *cognitive engagement*, and *agentic engagement* could be regarded as the sole factor within these dimensions. Additionally, we performed the Confirmatory Factor Analysis to exam the construct validity, and the fit indicators supported the validity with measures greater than 0.9 (Comparative Fit Index: 0.92; Normed Fit Index: 0.92; Non-Normed Fit Index: 0.90), with SRMR (0.053) performed well. In the following data analysis, we used the factor score coefficients to represent each student engagement factor. We provided the correlations among the factors of student engagement in the supplementary material (ST2). There are significant correlations between different student engagement factors.

We performed an empirical method of multivariate regression analyses to answer the research questions. Firstly, we regressed the scores of the Basic Medical Knowledge Examination and the Basic Clinical Skills Examination for each student engagement separately. We included student characteristics as control variables and included medical school fixed effects to control for all school-level factors in our cross-sectional data that may influence the outcomes. Moreover, to exclude the mutual correlations between the engagements, we included all the engagements in the regression simultaneously, so as to clarify the association between each engagement and learning outcomes after controlling other engagements. We were aware of the risk of multicollinearity and examined the Variance Inflation Factor when doing the analysis. Secondly, we replaced dependent variables in the first research question with the scores of memory, comprehension, and application parts in the Basic Medical Knowledge Examination, so as to examine the relationship between each student engagement factor and the cognitive levels of learning outcomes. Thirdly, we added gender and NCEE as two interaction terms to the regression to investigate whether gender and NCEE would affect the correlation between engagements and learning outcomes. All analyses were conducted using R and we regarded the differences to be significant if the p-value was < 0.01.

## Results

### Relationship between student engagement dimensions and learning outcomes

The results show that *emotional engagement* is positively related to learning outcomes in both the Basic Medical Knowledge Examination (β = 0.116, *p* < 0.001) and the Basic Clinical Skills Examination (β = 0.065, *p* < 0.001). *Cognitive engagement* positively correlate to students’ learning outcomes in the Basic Medical Knowledge Examination (β = 0.098, *p* < 0.001). However, to our surprise, *behavioral clinical engagement*, *behavioral classroom engagement*, and *agentic engagement* are negatively related to students’ learning outcomes in the Basic Medical Knowledge Examination (β=-0.077, *p* < 0.001; β=-0.023, *p* < 0.001; β=-0.061, *p* < 0.001 respectively). Meanwhile, we notice that the coefficients of these three student engagements change dramatically from separate inclusion to simultaneous inclusion (from model 1, 2, 5 to model 6), which indicates the significant correlations among these student engagements. The detailed results for the multivariate regression analyses can be found in Table 2.

**Table 2.** Student engagement and learning outcomes in the Basic medical Knowledge Examination and in the Basic clinical Skills Examination.

	Dependent variable: Learning outcomes in the Basic Medical Knowledge Examination						Dependent variable: Learning outcomes in the Basic Clinical Skills Examination					
	Model 1	Model 2	Model 3	Model 4	Model 5	Model 6	Model 1	Model 2	Model 3	Model 4	Model 5	Model 6
Behavioral clinical engagement	−0.034*** (0.009)					−0.077*** (0.009)	0.014 (0.008)					−0.012 (0.009)
Behavioral classroom engagement		0.023** (0.008)				−0.023** (0.009)		0.047*** (0.008)				0.019* (0.009)
Emotional engagement			0.128*** (0.008)			0.116*** (0.010)			0.084*** (0.008)			0.065*** (0.010)
Cognitive engagement				0.096*** (0.008)		0.098*** (0.014)				0.070*** (0.008)		0.023 (0.014)
Agentic engagement					0.032*** (0.008)	−0.061*** (0.012)					0.051*** (0.008)	0.001 (0.012)
Controls	Yes	Yes	Yes	Yes	Yes	Yes	Yes	Yes	Yes	Yes	Yes	Yes
School FE	Yes	Yes	Yes	Yes	Yes	Yes	Yes	Yes	Yes	Yes	Yes	Yes
R^2^	0.231	0.231	0.246	0.239	0.231	0.254	0.247	0.249	0.254	0.252	0.250	0.255
Adjusted R^2^	0.228	0.227	0.242	0.235	0.228	0.250	0.244	0.246	0.250	0.248	0.246	0.251
Observations	13010	13010	13010	13010	13010	13010	13010	13010	13010	13010	13010	13010

Notes: The learning outcomes were standardized among all students who participated in the test and the survey to obtain a mean of zero and a standard deviation of one. Standard errors in parentheses. ***p < 0.001, **0.001<*p* < 0.01, *0.01<*p* < 0.05. The controls contain gender, residence, father’s education duration, mother’s education duration, father’s occupation, mother’s occupation, whether having medical worker(s) in the family, and National College Entrance Examination (NCEE) result. Medical school fixed effects are included in all regressions. In the two Model 6, Variance Inflation Factors are all less than 5.

### Relationship between student engagement dimensions and learning outcomes of the memorization, comprehension, and application

We report how student engagement dimensions relate to learning outcomes in memorization, comprehension, and application in Table 3. Taking the categories of cognitive education objectives into consideration, we observe that *emotional engagement* and *cognitive engagement* are positively associated with learning outcomes in *memorization* (β = 0.101, *p* < 0.001; β = 0.098, *p* < 0.001), *comprehension* (β = 0.109, *p* < 0.001; β = 0.089, *p* < 0.001), and *application* (β = 0.115, *p* < 0.001; β = 0.094, *p* < 0.001). Among these, *emotional engagement* has the strongest correlation with all three categories of learning outcomes, followed by cognitive engagement. Whereas *behavioral clinical engagement* and *agentic engagement* show negative correlations with *memorization* (β=-0.071, *p* < 0.001; β=-0.063, *p* < 0.001), *comprehension* (β=-0.067, *p* < 0.001; β=-0.054, *p* < 0.001), and *application* (β=-0.076, *p* < 0.001; β=-0.057, *p* < 0.001). Moreover, *behavioral classroom engagement* is negatively associated with learning outcomes in *comprehension* (β=-0.029, *p* < 0.001).

**Table 3.** Relationship between student engagement and learning outcomes in memorization, comprehension, and application.

	Memorization	Comprehension	Application
	Model 1	Model 2	Model 3
Behavioral clinical engagement	−0.071*** (0.009)	−.067*** (.009)	−.076*** (.009)
Behavioral classroom engagement	−0.018* (0.009)	−.029*** (.009)	−.019* (.009)
Emotional engagement	0.101*** (0.011)	.109*** (.011)	.115*** (.010)
Cognitive engagement	0.098*** (0.014)	.089*** (.014)	.094*** (.014)
Agentic engagement	−0.063*** (0.012)	−.054*** (.012)	−.057*** (.012)
*Controls*	Yes	Yes	Yes
*School FE*	Yes	Yes	Yes
R^2^	0.210	.225	.249
Adjusted R^2^	0.206	.221	.245
Observations	13010	13010	13010

Notes: The learning outcomes were standardized among all students who participated in the test and the survey to obtain a mean of zero and a standard deviation of one. Standard errors in parentheses. ***p < 0.001, **0.001<*p* < 0.01, *0.01<*p* < 0.05. The controls contain gender, residence, father’s education duration, mother’s education duration, father’s occupation, mother’s occupation, whether having medical worker(s) in the family, and National College Entrance Examination (NCEE) result. Medical school fixed effects are included in all regressions. In Model 1–3, Variance Inflation Factors are all less than 5.

### Heterogeneity of students’ gender and NCEE result in relationships between student engagement and learning outcomes

After rigorous examination, we observe that gender (Table 4) does not influence the relationship between student engagement and learning outcomes. Whereas students’ NCEE results positively moderate the relationships between emotional engagement (β = 0.037, *p* < 0.001) and cognitive engagement (β = 0.028, *p* < 0.001) and the learning outcomes in the Basic Medical Knowledge Examination (Table 5). It indicates that students with higher NCEE results will gain more positive learning outcomes in the Basic Medical Knowledge Examination when engaging emotionally and cognitively. Nevertheless, neither gender nor NCEE plays a significant moderating role in the relationships in-between student engagement and learning outcomes in the Basic Clinical Skills Examination. We also provide the moderating effect of gender and NCEE results on relationships between student engagement and the cognitive levels of learning outcomes of the memorization, comprehension, and application in the Supplementary material (ST3 & ST4).

**Table 4.** Moderating effects of gender.

	Learning outcomes in the Basic Medical Knowledge Examination					Learning outcomes in the Basic Clinical Skills Examination				
	Model 1	Model 2	Model 3	Model 4	Model 5	Model 1	Model 2	Model 3	Model 4	Model 5
Behavioral clinical engagement	−0.078*** (0.011)	−0.077*** (0.009)	−0.077*** (0.009)	−0.077*** (0.009)	−0.077*** (0.009)	−0.008 (0.011)	−0.012 (0.009)	−0.012 (0.009)	−0.012 (0.009)	−0.012 (0.009)
Behavioral classroom engagement	−0.023** (0.009)	−0.027* (0.011)	−0.023** (0.009)	−0.023** (0.009)	−0.022** (0.009)	0.019* (0.009)	0.008 (0.011)	0.019* (0.009)	0.019* (0.009)	0.019* (0.009)
Emotional engagement	0.116*** (0.010)	0.116*** (0.010)	0.103*** (0.013)	0.116*** (0.010)	0.116*** (0.010)	0.066*** (0.010)	0.065*** (0.010)	0.050*** (0.013)	0.065*** (0.010)	0.066*** (0.010)
Cognitive engagement	0.098*** (0.014)	0.098*** (0.014)	0.098*** (0.014)	0.101*** (0.016)	0.098*** (0.014)	0.023 (0.014)	0.023 (0.014)	0.022 (0.014)	0.013 (0.016)	0.023 (0.014)
Agentic engagement	−0.061*** (0.012)	−0.061*** (0.012)	−0.061*** (0.012)	−0.061*** (0.012)	−0.050*** (0.014)	0.001 (0.012)	0.001 (0.012)	0.001 (0.012)	0.001 (0.012)	0.003 (0.014)
Male	−0.225*** (0.015)	−0.225*** (0.015)	−0.225*** (0.015)	−0.225*** (0.015)	−0.225*** (0.015)	−0.365*** (0.015)	−0.365*** (0.015)	−0.365*** (0.015)	−0.366*** (0.015)	−0.366*** (0.015)
Behavioral clinical engagement* Male	0.002 (0.015)					−0.008 (0.015)				
Behavioral classroom engagement* Male		0.009 (0.015)					0.024 (0.015)			
Emotional engagement *Male			0.028 (0.015)					0.034* (0.015)		
Cognitive engagement *Male				−0.006 (0.015)					0.022 (0.015)	
Agentic engagement *Male					−0.023 (0.015)					−0.003 (0.015)
Controls	Yes	Yes	Yes	Yes	Yes	Yes	Yes	Yes	Yes	Yes
School FE	Yes	Yes	Yes	Yes	Yes	Yes	Yes	Yes	Yes	Yes
R^2^	0.254	0.254	0.254	0.254	0.254	0.255	0.255	0.255	0.255	0.255
Adjusted R^2^	0.250	0.250	0.250	0.250	0.250	0.251	0.251	0.251	0.251	0.251
Observations	13010	13010	13010	13010	13010	13010	13010	13010	13010	13010

Notes: The controls contain residence, father’s education duration, mother’s education duration, father’s occupation, mother’s occupation, whether having medical worker(s) in the family, and National College Entrance Examination (NCEE) result. *** *p* < 0.001, ** 0.001<*p* < 0.01, *0.01<*p* < 0.05.

**Table 5.** Moderating effects of NCEE.

	Learning outcomes in the Basic Medical Knowledge Examination					Learning outcomes in the Basic Clinical Skills Examination				
	Model 1	Model 2	Model 3	Model 4	Model 5	Model 1	Model 2	Model 3	Model 4	Model 5
Behavioral clinical engagement	−0.077*** (0.009)	−0.076*** (0.009)	−0.076*** (0.009)	−0.076*** (0.009)	−0.076*** (0.009)	−0.012 (0.009)	−0.011 (0.009)	−0.011 (0.009)	−0.011 (0.009)	−0.012 (0.009)
Behavioral classroom engagement	−0.022** (0.009)	−0.023** (0.009)	−0.023** (0.009)	−0.023** (0.009)	−0.023** (0.009)	0.019* (0.009)	0.019* (0.009)	0.019* (0.009)	0.019* (0.009)	0.019* (0.009)
Emotional engagement	0.116*** (0.010)	0.116*** (0.010)	0.115*** (0.010)	0.117*** (0.010)	0.116*** (0.010)	0.065*** (0.010)	0.066*** (0.010)	0.065*** (0.010)	0.066*** (0.010)	0.066*** (0.010)
Cognitive engagement	0.099*** (0.014)	0.098*** (0.014)	0.099*** (0.014)	0.096*** (0.014)	0.098*** (0.014)	0.023 (0.014)	0.022 (0.014)	0.023 (0.014)	0.022 (0.014)	0.023 (0.014)
Agentic engagement	−0.061*** (0.012)	−0.061*** (0.012)	−0.061*** (0.012)	−0.061*** (0.012)	−0.062*** (0.012)	0.001 (0.012)	0.001 (0.012)	0.001 (0.012)	0.001 (0.012)	0.001 (0.012)
NCEE	0.209*** (0.010)	0.209*** (0.010)	0.211*** (0.010)	0.210*** (0.010)	0.209*** (0.010)	0.096*** (0.010)	0.096*** (0.010)	0.097*** (0.010)	0.097*** (0.010)	0.096*** (0.010)
Behavioral clinical engagement* NCEE	0.013 (0.008)					−0.004 (0.008)				
Behavioral classroom engagement* NCEE		0.020* (0.008)					0.016* (0.008)			
Emotional engagement* NCEE			0.037*** (0.007)					0.018* (0.007)		
Cognitive engagement * NCEE				0.028*** (0.007)					0.004 (0.007)	
Agentic engagement * NCEE					0.016* (0.007)					0.001 (0.007)
Controls	Yes	Yes	Yes	Yes	Yes	Yes	Yes	Yes	Yes	Yes
School FE	Yes	Yes	Yes	Yes	Yes	Yes	Yes	Yes	Yes	Yes
R^2^	0.254	0.254	0.255	0.254	0.254	0.255	0.255	0.255	0.255	0.255
Adjusted R^2^	0.250	0.250	0.251	0.250	0.250	0.251	0.251	0.251	0.251	0.251
Observations	13010	13010	13010	13010	13010	13010	13010	13010	13010	13010

Notes: The controls contain gender, residence, father’s education duration, mother’s education duration, father’s occupation, mother’s occupation, and whether having medical worker(s) in the family. *** *p* < 0.001, ** 0.001<*p* < 0.01, *0.01<*p* < 0.05.

**Figure 1.**
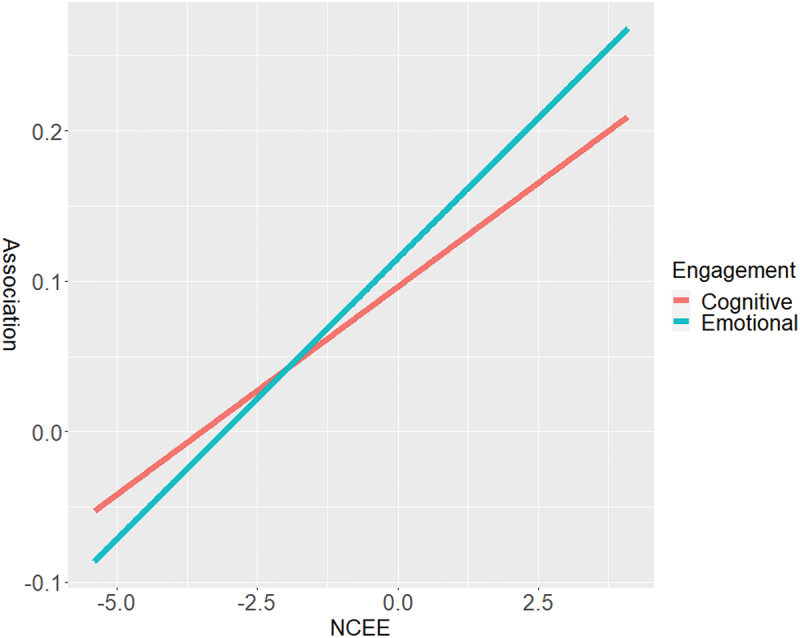
Post-hoc probing for the moderating effects of NCEE.

Notes: The figure displays the moderating effects of NCEE based on the estimations from Model 3 and Model 4 in Table 5. The x-axis represents the standardized score on NCEE, while the y-axis represents the association between the Basic Medical Knowledge Examination and the two student engagements.

Furthermore, we have conducted post-hoc probing to provide additional potential explanations for the moderating effects of NCEE. We used Figure 1 to visualize the moderating effects of NCEE according to the estimations of Model 3 (NCEE interacts with Emotional engagement) and Model 4 (NCEE interacts with Cognitive engagement) in Table 5. In Figure 1, the x-axis represents the standardized score on NCEE, and the y-axis represents the association between the Basic Medical Knowledge Examination and the two student engagements. It was found that as the NCEE score increase, the association between the Basic Medical Knowledge Examination and these two engagements also increases correspondingly. The increased slope represents the interaction coefficient between the two student engagements and the Basic Medical Knowledge Examination in the regression, which is 0.037 (Emotional engagement) and 0.028 (Cognitive engagement) respectively. In other words, for every 1 standard deviation increase in NCEE scores, the association between Basic Medical Knowledge and emotional engagement increases by 0.037, and the association between Basic Medical Knowledge and cognitive engagement increases by 0.028.

## Discussion

This study applies Reeve’s four-dimensional student engagement framework to a medical education context to elucidate the relationship between behavioral, emotional, cognitive, and agentic engagement and learning outcomes. Through the use of nationwide data and the combination of student engagement with objectively assessed learning outcomes, we have delved into the effectiveness of different types of student engagement.

Unexpectedly, behavioral clinical engagement and behavioral classroom engagement showed a negative correlation with the learning outcomes. Although behavioral engagement has been shown to be a robust predictor of achievement in educational research [20,42], it highly depends on the types of assessment that have been used in the study [12,13]. Behavioral engagement showed good effectiveness when the assessments were low-level tests based on simple recall of attendance [13], and the achievements where behavioral engagement was shown to be effective were normally content based on a micro level (such as course tests) [42,43]. Whereas for exams requiring higher-order processing strategies, behavioral engagement may not be a good predictor [13]. Meanwhile, the negative correlation between behavioral engagement and learning outcomes in this study could indicate the distance between the medical training in schools and the Proficiency Test. In other words, there might be a mismatch between what students learn in the curriculum and what is tested at the general competency test, and, accordingly, the behavioral engagement in the curriculum may not be sufficient to prepare students to take the test. Consistent with other findings that students may need extra preparation using third-party study resources to prepare for the USMILE [44,45]. Such mismatches between curriculum design and general competency tests may also exist in other higher education contexts.

In line with former research, this study provides evidence for the effectiveness of emotional engagement in multiple components of learning outcomes. Students who showed higher interest and enthusiasm in what they were learning tend to get better learning outcomes and satisfaction [9]. Higher levels of emotional engagement likewise represent higher levels of intrinsic motivation, which may result in students contributing high task values throughout the learning process [13]. In medical education, medical students’ intentions and enthusiasm for work closely related to their profession, such as becoming a medical doctor, make emotional engagement a good predictor of macro-level learning outcomes assessment. Although academic interests may play a more important role in academic success in medical science than in some other disciplines such as the humanities or social sciences, they are not very different from other disciplines such as the natural sciences and engineering programs [46]. Thus, the results of this study could suggest for general higher education that learning outcomes could be significantly enhanced when students are interested and enthusiastic about what they are learning, or when the learning activities from the institutions could stimulate students’ emotional engagement.

This study also indicates that in medical education content and beyond, cognitive engagement could help students with better memorization, comprehension, and application of the target knowledge. It is recognized that motivational and/or self-regulatory constructs are present in every dimension of student engagement, and this is especially true for cognitive engagement [13,21]. Cognitive engagement encompasses not only the shallow-level processing of the learning process but also the deep-level processing [21].

Next, our evidence does not demonstrate that agentic engagement promotes learning outcomes in the Chinese medical education context. Agentic engagement refers to dynamic teaching and learning processes in which students not only passively engage, but also provide evaluation and feedback on the learning process [8,18]. This places high demands on the learning environment and institutions need to be prepared to build bi-directional interactions and take into account student feedback in the curriculum design process [8,18]. Besides, it could also partially be explained by the earlier mentioned gap between the curriculum and the Proficiency Test because students’ feedback towards the learning activities is also mostly instantaneous [47]. The results of this study could not point to a limited contribution of agentic engagement to learning outcomes; rather, the results reflect that in China the undergraduate medical curricula are not adequately prepared for learning environments in which students could be agentic engaged. Another explanation may lie in the cultural traits. Chinese culture is recognized as having a relatively high power distance, where students may be reluctant to criticize the curriculum or to proactively provide advice to teachers or institutions [48]. Furthermore, whilst agentic engagement has been proposed as a student-initiated pathway for better achievement and greater motivational support [17], it is possible that in practice students with unsatisfactory academic performance are keener to provide advice to institutions. For medical classrooms in China with large class sizes in particular, students who are dissatisfied with their academic performance may have a higher motivation to offer suggestions to their teachers. Additional research on creating learning environments that enable agentic engagement in different contexts is certainly needed.

Moreover, the positive moderating effect of the NCEE aligns with previous research that high school achievements matter when trying to explain the relationship between university achievements and student engagement [6,11,38]. The NCEE result, as a representation of high school performance, has been shown to correlate positively with students’ university performance and can be used as a consistent indicator of students’ academic ability [39]. Thus, students with higher NCEE scores are likely to have better academic abilities pertaining to concentration ability, time management skills, self-determined learning capacity, and greater enthusiasm for what they are learning. These characteristics enable students with higher NCEE scores to achieve high-quality student engagement, particularly in terms of emotional engagement and cognitive engagement, which ensures better learning outcomes for them.

### Limitations and directions for future research

We acknowledge certain limitations in this study. Firstly, although we avoided using self-reported data for measuring learning outcomes, the student engagement measurements were still self-reported. Students recall their extent of student engagement in learning activities, which leaves some of the problems inherent in self-report measures unavoidable. Secondly, we have selected our own student engagement items from the existing questionnaire in the context of medical education in China considering both content and structure. Instead of using the original items from Reeve [17], the items in our study are integrated with the Chinese medical education context and the guidance from existing research. The external validity of the results of this study in the context of other countries or different disciplines needs to be concerned. It will be interesting for future research to explore the effectiveness of the four student engagement dimensions in different cultural and disciplinary contexts using a more systematic approach to item design. Thirdly, this study did not further examine how different student engagement dimensions interacted with each other. Future research could therefore investigate how each student engagement dimension moderates the other student engagement dimensions and learning outcomes. Furthermore, although we have added various controls including gender, residence, parents’ education duration, parents’ occupation ISEI, whether having medical worker(s) in the family, and NCEE result, and added Medical school as fixed effects, we might be still missing other variables that may influence the results such as cultural value, whether studying medicine is the first choice, and class size. Future studies could consider more control variables to ensure the accuracy of the results.

### Implications for higher educational practice

The results of our study suggest that emotional engagement and cognitive engagement could be used as good predictors of learning outcomes in macro-level general competence tests, whereas behavioral and agentic engagement may not. Moreover, more efforts need to be put into agentic engagement to figure out what constitutes a conducive learning environment that allows for bidirectional interactions between students and institutions alongside the learning process.

For practical implications, the negative correlations of behavioral engagement and agentic engagement on learning outcomes enlighten us to rethink the distance between general competency tests and daily learning activities. It is necessary to bridge the distance, allowing students to acquire what is tested in the general competency test from their daily learning. Alternatively, the general competency test could be brought closer to the knowledge and skills that students acquire from their learning activities. Communication between curriculum providers and examination development organizations should be strengthened to ensure that the general competency tests are up-to-date in alignment with the curriculum content. Besides, this study indicates that students need to be more self-conscious about the effectiveness of student engagement. Being interested, passionate, and self-determined in their professional field could drive better learning outcomes for students. Meanwhile, the institutions need to prepare a learning environment with well-designed learning activities that encourage student engagement and enable good interaction between students and the institution.

## Conclusion

This study juxtaposes behavioral, emotional, cognitive, and agentic engagement together, uses Chinese national-wide data and learning outcomes from standardized examination, and provides robust evidence for the effectiveness of emotional engagement and cognitive engagement in promoting learning outcomes. By contrast, behavioral engagement and agentic engagement may not be good predictors of learning outcomes in macro-level general competence tests. This study enlightens us to strengthen the communication between curriculum providers and examination development organizations.

## Supplementary Material

Supplemental Material
