# Supplementary material for: Student engagement and learning outcomes: an empirical study applying a four-dimensional framework
Source: Med Educ Online. 2023 Oct 8;28(1):2268347. doi: 10.1080/10872981.2023.2268347 (PMC10563621; doi:10.1080/10872981.2023.2268347)
Supplement: Supplemental Material [file ZMEO_A_2268347_SM2726.docx]

**ST1** Sample items in the Proficiency test

| **Basic Medical Knowledge Examination** |  |
| --- | --- |
| Memorization | The most common opportunistic infections in people with HIV is (Single-choice):  **A.** Cryptococcal meningitis; **B.** Herpes zoster; **C.** Candida pneumonia; **D.** Pneumocystis pneumonia; **E.** Cytomegalovirus esophagitis |
| Comprehension | A medical researcher uses a drug in the experimental stage for clinical use and charges patients for it. The main ethical requirements of medical research that this practice violates is (Single-choice):  **A.** Courage to innovate; **B.** Standardization of operations; **C.** Improvements in excellence; **D.** Purity of motivation; **E.** Public-Private Balance |
| Application | Female, 35 years old, progressive limbs weakness for 1 year, mild in the morning, aggravated in the afternoon. There was a history of pectoral adenoma disease, denying the history of hyperthyroidism. Positive exercise fatigue test. At present, the clinical consideration is severe muscle weakness, and the symptoms were relieved by cholinesterase inhibitors, but the most likely new symptoms is (Single-choice):  **A.** Diarrhea; **B.** Tachycardia; **C.** Dilated pupils; **D.** Dry mouth; **E.** Breathing difficulties |
| **Basic Clinical Skills Examination** |  |
|  | Medical history taking for fever (Ten-minute interview with standardized patients) |
|  | Physical examination of the general condition (Ten-minute interview with standard medical examiners) |
|  | Basic operative skills for basic surgical operations (Ten-minute interview with operating on medical teaching simulators or medical models) |

**ST2** Correlation coefficients of student engagement dimensions

| Construct | 1 | 2 | 3 | 4 | 5 | 6 | 7 | 8 | 9 | 10 |
| --- | --- | --- | --- | --- | --- | --- | --- | --- | --- | --- |
| 1. Behavioral clinical engagement | 1.00 |  |  |  |  |  |  |  |  |  |
| 1. Behavioral classroom engagement | -- | 1.00 |  |  |  |  |  |  |  |  |
| 1. Emotional engagement | 0.22*** | 0.29*** | 1.00 |  |  |  |  |  |  |  |
| 1. Cognitive engagement | 0.30*** | 0.36*** | 0.66*** | 1.00 |  |  |  |  |  |  |
| 1. Agentic engagement | 0.30*** | 0.37*** | 0.45*** | 0.76*** | 1.00 |  |  |  |  |  |
| 1. Basic Medical Knowledge Examination | -0.02* | 0.04*** | 0.14*** | 0.11*** | 0.03** | 1.00 |  |  |  |  |
| 1. Basic Clinical Skills Examination | -0.02* | 0.06*** | 0.11*** | 0.09*** | 0.07*** | 0.48*** | 1.00 |  |  |  |
| 1. Memorization | -0.02** | 0.04*** | 0.13*** | 0.10*** | 0.02** | 0.92*** | 0.43*** | 1.00 |  |  |
| 1. Comprehension | -0.02* | 0.03*** | 0.13*** | 0.10*** | 0.03** | 0.93*** | 0.45*** | 0.82*** | 1.00 |  |
| 1. Application | -0.02 | 0.04*** | 0.14*** | 0.11*** | 0.03** | 0.97*** | 0.47*** | 0.84*** | 0.85*** | 1.00 |

*** p<0.001, ** 0.001<p<0.01, * 0.01<p<0.05

**ST3** Moderating effects of gender on the cognitive levels of learning outcomes

|  | Learning outcomes -- Memorization | | | | | Learning outcomes -- Comprehension | | | | | Learning outcomes -- Application | | | | |
| --- | --- | --- | --- | --- | --- | --- | --- | --- | --- | --- | --- | --- | --- | --- | --- |
|  | Model 1 | Model 2 | Model 3 | Model 4 | Model 5 | Model 1 | Model 2 | Model 3 | Model 4 | Model 5 | Model 1 | Model 2 | Model 3 | Model 4 | Model 5 |
| Behavioral clinical engagement | -0.080^***^ | -0.072^***^ | -0.072^***^ | -0.071^***^ | -0.071^***^ | -0.072^***^ | -0.067^***^ | -0.068^***^ | -0.067^***^ | -0.067^***^ | -0.071^***^ | -0.076^***^ | -0.076^***^ | -0.075^***^ | -0.075^***^ |
|  | (0.011) | (0.009) | (0.009) | (0.009) | (0.009) | (0.011) | (0.009) | (0.009) | (0.009) | (0.009) | (0.011) | (0.009) | (0.009) | (0.009) | (0.009) |
| Behavioral classroom engagement | -0.018^*^ | -0.023^*^ | -0.018^*^ | -0.018^*^ | -0.018^*^ | -0.029^***^ | -0.032^**^ | -0.030^***^ | -0.029^***^ | -0.029^***^ | -0.019^*^ | -0.024^*^ | -0.019^*^ | -0.019^*^ | -0.019^*^ |
|  | (0.009) | (0.012) | (0.009) | (0.009) | (0.009) | (0.009) | (0.012) | (0.009) | (0.009) | (0.009) | (0.009) | (0.011) | (0.009) | (0.009) | (0.009) |
| Emotional engagement | 0.100^***^ | 0.101^***^ | 0.091^***^ | 0.101^***^ | 0.101^***^ | 0.108^***^ | 0.109^***^ | 0.099^***^ | 0.109^***^ | 0.109^***^ | 0.115^***^ | 0.115^***^ | 0.099^***^ | 0.115^***^ | 0.115^***^ |
|  | (0.011) | (0.011) | (0.013) | (0.011) | (0.011) | (0.011) | (0.011) | (0.013) | (0.011) | (0.011) | (0.010) | (0.010) | (0.013) | (0.010) | (0.010) |
| Cognitive engagement | 0.098^***^ | 0.098^***^ | 0.098^***^ | 0.105^***^ | 0.098^***^ | 0.088*** | 0.089*** | 0.088*** | 0.092*** | 0.089*** | 0.094*** | 0.094*** | 0.093*** | 0.094*** | 0.094*** |
|  | (0.014) | (0.014) | (0.014) | (0.016) | (0.014) | (0.014) | (0.014) | (0.014) | (0.016) | (0.014) | (0.014) | (0.014) | (0.014) | (0.016) | (0.014) |
| Agentic engagement | -0.063^***^ | -0.064^***^ | -0.063^***^ | -0.063^***^ | -0.048^**^ | -0.054^***^ | -0.054^***^ | -0.054^***^ | -0.054^***^ | -0.044^**^ | -0.057^***^ | -0.057^***^ | -0.057^***^ | -0.057^***^ | -0.049^***^ |
|  | (0.012) | (0.012) | (0.012) | (0.012) | (0.015) | (0.012) | (0.012) | (0.012) | (0.012) | (0.014) | (0.012) | (0.012) | (0.012) | (0.012) | (0.014) |
| Male | -0.150^***^ | -0.149^***^ | -0.149^***^ | -0.149^***^ | -0.149^***^ | -0.145^***^ | -0.145^***^ | -0.145^***^ | -0.145^***^ | -0.145^***^ | -0.278^***^ | -0.278^***^ | -0.278^***^ | -0.278^***^ | -0.278^***^ |
|  | (0.016) | (0.016) | (0.016) | (0.016) | (0.016) | (0.016) | (0.016) | (0.016) | (0.016) | (0.016) | (0.016) | (0.016) | (0.016) | (0.016) | (0.016) |
| Behavioral clinical engagement* Male | 0.019  (0.016) |  |  |  |  | 0.010  (0.016) |  |  |  |  | -0.010  (0.015) |  |  |  |  |
| Behavioral classroom engagement* Male |  | 0.010  (0.016) |  |  |  |  | 0.005  (0.016) |  |  |  |  | 0.010  (0.015) |  |  |  |
| Emotional engagement*Male |  |  | 0.021  (0.016) |  |  |  |  | 0.020  (0.016) |  |  |  |  | 0.033^*^  (0.015) |  |  |
| Cognitive engagement*Male |  |  |  | -0.014  (0.016) |  |  |  |  | -0.006  (0.016) |  |  |  |  | -0.001  (0.015) |  |
| Agentic engagement*Male |  |  |  |  | -0.033^*^  (0.016) |  |  |  |  | -0.020  (0.016) |  |  |  |  | -0.018  (0.015) |
| Controls | Yes | Yes | Yes | Yes | Yes | Yes | Yes | Yes | Yes | Yes | Yes | Yes | Yes | Yes | Yes |
| School FE | Yes | Yes | Yes | Yes | Yes | Yes | Yes | Yes | Yes | Yes | Yes | Yes | Yes | Yes | Yes |
| R^2^ | 0.210 | 0.210 | 0.210 | 0.210 | 0.210 | 0.225 | 0.225 | 0.225 | 0.225 | 0.225 | 0.249 | 0.249 | 0.249 | 0.249 | 0.249 |
| Adjusted R^2^ | 0.206 | 0.206 | 0.206 | 0.206 | 0.206 | 0.221 | 0.221 | 0.221 | 0.221 | 0.221 | 0.245 | 0.245 | 0.245 | 0.245 | 0.245 |
| Observations | 13010 | 13010 | 13010 | 13010 | 13010 | 13010 | 13010 | 13010 | 13010 | 13010 | 13010 | 13010 | 13010 | 13010 | 13010 |

**Notes**: Standard errors in parentheses. *** p<0.001, ** 0.001<p<0.01, *0.01<p<0.05. The controls contain residence, father's education duration, mother's education duration, father’s occupation, mother’s occupation, whether having medical worker(s) in the family, and National College Entrance Examination (NCEE) result. Medical school fixed effects are included in all regressions.

**ST4** Moderating effects of NCEE on the cognitive levels of learning outcomes

|  | Learning outcomes -- Memorization | | | | | Learning outcomes -- Comprehension | | | | | Learning outcomes -- Application | | | | |
| --- | --- | --- | --- | --- | --- | --- | --- | --- | --- | --- | --- | --- | --- | --- | --- |
|  | Model 1 | Model 2 | Model 3 | Model 4 | Model 5 | Model 1 | Model 2 | Model 3 | Model 4 | Model 5 | Model 1 | Model 2 | Model 3 | Model 4 | Model 5 |
| Behavioral clinical engagement | -0.071^***^ | -0.071^***^ | -0.071^***^ | -0.071^***^ | -0.071^***^ | -0.067^***^ | -0.067^***^ | -0.067^***^ | -0.067^***^ | -0.067^***^ | -0.075^***^ | -0.075^***^ | -0.075^***^ | -0.074^***^ | -0.075^***^ |
|  | (0.009) | (0.009) | (0.009) | (0.009) | (0.009) | (0.009) | (0.009) | (0.009) | (0.009) | (0.009) | (0.009) | (0.009) | (0.009) | (0.009) | (0.009) |
| Behavioral classroom engagement | -0.018^*^ | -0.019^*^ | -0.019^*^ | -0.019^*^ | -0.018^*^ | -0.029^***^ | -0.030^***^ | -0.030^***^ | -0.030^***^ | -0.030^***^ | -0.019^*^ | -0.020^*^ | -0.019^*^ | -0.019^*^ | -0.019^*^ |
|  | (0.009) | (0.009) | (0.009) | (0.009) | (0.009) | (0.009) | (0.009) | (0.009) | (0.009) | (0.009) | (0.009) | (0.009) | (0.009) | (0.009) | (0.009) |
| Emotional engagement | 0.101^***^ | 0.101^***^ | 0.100^***^ | 0.102^***^ | 0.101^***^ | 0.109^***^ | 0.109^***^ | 0.108^***^ | 0.109^***^ | 0.109^***^ | 0.115^***^ | 0.115^***^ | 0.114^***^ | 0.116^***^ | 0.115^***^ |
|  | (0.011) | (0.011) | (0.011) | (0.011) | (0.011) | (0.011) | (0.011) | (0.011) | (0.011) | (0.011) | (0.010) | (0.010) | (0.013) | (0.010) | (0.010) |
| Cognitive engagement | 0.098^***^ | 0.098^***^ | 0.099^***^ | 0.097^***^ | 0.098^***^ | 0.089^***^ | 0.088^***^ | 0.089^***^ | 0.087^***^ | 0.089^***^ | 0.094*** | 0.093*** | 0.094*** | 0.092*** | 0.094*** |
|  | (0.014) | (0.014) | (0.014) | (0.014) | (0.014) | (0.014) | (0.014) | (0.014) | (0.014) | (0.014) | (0.014) | (0.014) | (0.014) | (0.014) | (0.014) |
| Agentic engagement | -0.064^***^ | -0.063^***^ | -0.064^***^ | -0.063^***^ | -0.064^***^ | -0.054^***^ | -0.054^***^ | -0.054^***^ | -0.054^***^ | -0.055^***^ | -0.058^***^ | -0.057^***^ | -0.058^***^ | -0.057^***^ | -0.059^***^ |
|  | (0.012) | (0.012) | (0.012) | (0.012) | (0.012) | (0.012) | (0.012) | (0.012) | (0.012) | (0.012) | (0.012) | (0.012) | (0.012) | (0.012) | (0.012) |
| NCEE | 0.220^***^ | 0.219^***^ | 0.221^***^ | 0.220^***^ | 0.220^***^ | 0.221^***^ | 0.221^***^ | 0.222^***^ | 0.222^***^ | 0.221^***^ | -0.178^***^ | -0.178^***^ | -0.180^***^ | -0.179^***^ | -0.178^***^ |
|  | (0.011) | (0.011) | (0.011) | (0.011) | (0.011) | (0.011) | (0.011) | (0.011) | (0.011) | (0.011) | (0.010) | (0.010) | (0.010) | (0.010) | (0.010) |
| Behavioral clinical engagement* NCEE | 0.008  (0.008) |  |  |  |  | 0.014  (0.008) |  |  |  |  | 0.014  (0.008) |  |  |  |  |
| Behavioral classroom engagement* NCEE |  | 0.016^*^  (0.008) |  |  |  |  | 0.015  (0.008) |  |  |  |  | 0.022^**^  (0.008) |  |  |  |
| Emotional engagement* NCEE |  |  | 0.032^***^  (0.008) |  |  |  |  | 0.028^***^  (0.008) |  |  |  |  | 0.040^***^  (0.007) |  |  |
| Cognitive engagement* NCEE |  |  |  | 0.024^**^  (0.008) |  |  |  |  | 0.018^*^  (0.008) |  |  |  |  | 0.031^***^  (0.007) |  |
| Agentic engagement* NCEE |  |  |  |  | 0.010  (0.008) |  |  |  |  | 0.013  (0.008) |  |  |  |  | 0.020^**^  (0.007) |
| Controls | Yes | Yes | Yes | Yes | Yes | Yes | Yes | Yes | Yes | Yes | Yes | Yes | Yes | Yes | Yes |
| School FE | Yes | Yes | Yes | Yes | Yes | Yes | Yes | Yes | Yes | Yes | Yes | Yes | Yes | Yes | Yes |
| R^2^ | 0.210 | 0.210 | 0.211 | 0.211 | 0.210 | 0.225 | 0.225 | 0.226 | 0.225 | 0.225 | 0.249 | 0.250 | 0.251 | 0.250 | 0.250 |
| Adjusted R^2^ | 0.206 | 0.206 | 0.207 | 0.207 | 0.206 | 0.221 | 0.221 | 0.221 | 0.221 | 0.221 | 0.246 | 0.246 | 0.247 | 0.246 | 0.246 |
| Observations | 13010 | 13010 | 13010 | 13010 | 13010 | 13010 | 13010 | 13010 | 13010 | 13010 | 13010 | 13010 | 13010 | 13010 | 13010 |

**Notes**: Standard errors in parentheses. *** p<0.001, ** 0.001<p<0.01, *0.01<p<0.05. The controls contain gender, residence, father's education duration, mother's education duration, father’s occupation, mother’s occupation, and whether having medical worker(s) in the family. Medical school fixed effects are included in all regressions.
